# Ultrastructural characteristics of oligodendrocyte precursor cells in the early postnatal mouse optic nerve observed by serial block-face scanning electron microscopy

**DOI:** 10.1371/journal.pone.0278118

**Published:** 2022-12-01

**Authors:** Katsuhiko Ono, Hitoshi Gotoh, Tadashi Nomura, Tsuyoshi Morita, Otto Baba, Mami Matsumoto, Sei Saitoh, Nobuhiko Ohno

**Affiliations:** 1 Developmental Neurobiology, Graduate School of Medical Science, Kyoto Prefectural University of Medicine, Kyoto, Japan; 2 Oral & Maxillofacial Anatomy, Graduate School of Oral Science, Tokushima University, Tokushima, Japan; 3 Section of Electron Microscopy, Supportive for Brain Research, National Institute for Physiological Sciences, Okazaki, Japan; 4 Developmental & Regenerative Neurobiology, Institute of Brain Science, Nagoya City University Graduate School of Medical Sciences, Nagoya, Japan; 5 Department of Anatomy II and Cell Biology, Fujita Health University, School of Medicine, Toyoake, Japan; 6 Division of Histology and Cell Biology, Department of Anatomy, Jichi Medical University School of Medicine, Tochigi, Japan; 7 Division of Ultrastructural Research, National Institute for Physiological Sciences, Okazaki, Japan; Federal University of Rio de Janeiro, BRAZIL

## Abstract

Oligodendrocyte precursor cells (OPC) arise from restricted regions of the central nervous system (CNS) and differentiate into myelin-forming cells after migration, but their ultrastructural characteristics have not been fully elucidated. This study examined the three-dimensional ultrastructure of OPCs in comparison with other glial cells in the early postnatal optic nerve by serial block-face scanning electron microscopy. We examined 70 putative OPCs (pOPC) that were distinct from other glial cells according to established morphological criteria. The pOPCs were unipolar in shape with relatively few processes, and their Golgi apparatus were localized in the perinuclear region with a single cisterna. Astrocytes abundant in the optic nerve were distinct from pOPCs and had a greater number of processes and more complicated Golgi apparatus morphology. All pOPCs and astrocytes contained a pair of centrioles (basal bodies). Among them, 45% of pOPCs extended a short cilium, and 20% of pOPCs had centrioles accompanied by vesicles, whereas all astrocytes with basal bodies had cilia with invaginated ciliary pockets. These results suggest that the fine structures of pOPCs during the developing and immature stages may account for their distinct behavior. Additionally, the vesicular transport of the centrioles, along with a short cilium length, suggests active ciliogenesis in pOPCs.

## Introduction

Oligodendrocytes (OL) are myelin-forming cells in the central nervous system (CNS), including the optic nerve [1]. OLs develop from oligodendrocyte precursor cells (OPC), which arise from the restricted region of the ventricular zone of the CNS in a stage-dependent manner [2–7]. OPCs were initially identified in cultured optic nerve cells of newborn rats as bipotential glial progenitor cells [8]. These bipotential glial progenitor cells proliferate in response to PDGF-A and express a receptor for PDGF-A (PDGFRα) [9]. When bipotential glial progenitor cells were transplanted into newborn rat brains, all grafted cells differentiated into myelinating OLs [10]. Thus, these bipotential glial progenitor cells were revealed to be equivalent to OPCs in vivo. In addition, it was found that PDGFRα can be a lineage marker for OPCs in vitro and in vivo [3]. Using this lineage marker along with various transgenic and knockout mice, the developmental processes of OLs and OPCs, as well as the molecular mechanisms underlying OPC development, have been extensively studied in vivo [11–14]. Numerous studies have elucidated that OPCs commit to OL lineage and express NG2 proteoglycan, as well as PDGFRα, and some of them persist in the adult CNS [15–17].

The ultrastructure of nerve cells has been widely studied by transmission electron microscopy (TEM) until the 1980s [18,19]. TEM studies revealed the ultrastructural characteristics of four major cell types in the mature CNS. First, neurons are identified by the presence of synaptic contacts on cell bodies and processes. Second, astrocytes are identified as cells containing bundles of intermediate filaments and glycogen granules and show irregular contour-forming thin membranous processes that intercalate narrow neuron-neuron spaces. Third, OLs have an electron-dense dark cytoplasm and are stacked in the rough endoplasmic reticulum (ER). Fourth, microglia have dense bodies, namely lysosomes and lipofuscin, in the cytoplasm. The TEM studies in those days paid little attention OPCs because they had not been identified either in vitro or in vivo. After the identification of OPCs, it was found that they express several ion channels, including glutamate receptor channels of the quisqualate and kinate types, and that the cells expressing these ion channels take up extracellular cobalt, allowing histochemical visualization of the cells. This histochemical technique was applied to identify OPCs by TEM and revealed that OPCs did not exhibit any of the characteristic features of differentiated glial cells [20]. Notably, individual TEM pictures only show a single plane of cells, and it is often difficult to identify the cell types if the single plane does not contain typical features. In addition, it is difficult to understand the complex and rare structures of the cells using only a single plane and focusing on typical features. Therefore, the ultrastructural characteristics of OPCs have not been fully clarified.

In the present study, we scrutinized the ultrastructural characteristics of OPCs in the newborn mouse optic nerve. We utilized a novel approach known as serial block-face scanning electron microscopy (SBEM or SBF-SEM), which acquires serial electron microscopic images from single cells and captures the whole cell ultrastructure from one side of the cell to the opposite end at the subcellular level [21,22]. Using the SEM technology, we reconstructed three-dimensional (3D) structures of OPCs and other glial cells from serial 2D images [23], enabling the identification of cell types in the CNS and the understanding of their 3D subcellular structures. We applied this approach to the optic nerve on postnatal day 4 (P4), which has the following advantages: (i) P4 is the stage before myelination starts, and therefore, there are no myelinating OLs, and (ii) the P4 optic nerve is composed of only glial cells, including astrocytes, microglia, and OPCs or glial progenitor cells. The results demonstrated that the 3D fine structures of OPCs in the P4 optic nerve were distinct from those of astrocytes. The OPCs had relatively few processes and ER, and immature cilium and basal bodies, compared with astrocytes. In addition, the Golgi apparatus and cell contours were more complicated in astrocytes than in OPCs.

## Materials and methods

### Animals and tissue preparation

The pregnant dams of mice with newborn pups (ICR strain) were purchased from Shizuoka Laboratory Animal Center (Hamamatsu, Japan). From two dams, the P4 mice from two dams were euthanized by injection of a lethal dose of sodium pentobarbital. The animals were quickly perfused through the heart with a mixed solution of 2% paraformaldehyde (PFA) and 2.5% glutaraldehyde in phosphate-buffered saline (PBS) to fix the optic nerve for SBF-SEM. We used two optic nerves for SBF-SEM analysis. For immunohistochemistry, from three dams, P2 and P4 pups were euthanized and perfused for fixation with 4% PFA. All animal experiment procedures were approved by the Animal Research Committee of the Kyoto Prefectural University of Medicine.

### SBF-SEM observations

For SBF-SEM, the optic nerve tissues were prepared as previously described, with some modifications [24,25]. The optic nerves were removed from the skull base, and the middle part of the optic nerve, not the chiasmal or retinal side, was used for analysis. The optic nerve tissues were immersed in the same fixative overnight at 4°C. They were then post-fixed with a mixed solution of 1.5% potassium ferrocyanide and 2% OsO_4_ in PBS for 1 hour on ice, followed by 1% thiocarbohydrazide in distilled water for 20 min, then 2% OsO_4_ for 30 min at room temperature. The samples were stained en bloc with 2% uranyl acetate overnight, followed by lead aspartate solution for 30 min at 65°C. The optic nerves were dehydrated using a graded series of ethanol, infiltrated with acetone, and embedded in Quetol 812 epoxy resin containing Ketjen black powder.

SBF-SEM of the optic nerves was performed using a Merlin and Sigma scanning electron microscope (Carl Zeiss) equipped with a 3View in-chamber ultramicrotome system (Gatan). Sequential images were processed using Fiji software (NIH) to convert images from DM3 to Tiff format and reduce the file size. Segmentation of the cell bodies, nuclei, centrioles, Golgi apparatus, ER, and cilia was performed using Microscopy Image Browser (http://mib.helsinki.fi/) [26]. Manual segmentation was done in every 2nd–10th slice and an interpolation tool was used for the other slices. Reconstructions of glial cells and their organelles were performed using Amira software (Thermo Fischer Scientific). Six astrocytes and 11 OPCs were three-dimensionally reconstructed, along with their nuclei and cytoplasmic organelles, as mentioned above. After 3D reconstruction, the surface areas and volumes were analyzed for the whole cells. The surface areas and volumes of Golgi apparatus and whole cells, and lengths of cilia, were also measured using Amira software. Some cilia and Golgi apparatus were three-dimensionally reconstructed independently of cell bodies. The complexity of the surface profile of cells and Golgi apparatus was expressed as a ratio of surface area to volume.

### Immunohistochemistry

The optic nerves were cut longitudinally with 20-μm thicknesses using a cryostat (Leica CM1850). They were incubated with primary antibodies overnight and subsequently with species-specific secondary antibodies conjugated to Alexaflour 488 or 594 (Thermo Fischer Science, 1:1000) for 40 min. Cell nuclei were stained with Hoechst33342 (Nacalai Tesque, Kyoto, Japan, 1 μg/μL) or TO-PRO (Thermo Fischer Science, 1:100000). We used the following primary antibodies: goat anti-PDGFRα (R&D systems, Minneapolis, MN, AF1062, 1:1000), rabbit anti-Iba-1 (Wako Fuji film, 1:1000), guinea pig anti-GLAST (Frontier Science Inst. Hakodate, Japan, Af1000, 1:1000), mouse IgM anti-glycogen (culture supernatant) [27,28], mouse-anti γ-tubulin (Sigma-Aldrich, St. Louis, T-6557, 1:500). The sections were observed using an epifluorescent microscope (Olympus, BX-50) and a confocal laser scanning microscope (Olympus CLSM, FV-1000).

### Statistical analysis

The data were presented as the mean ± S.D. The results were evaluated by one-way analysis of variance and using unpaired t-tests and Mann–Whitney U tests with Microsoft Excel software (Microsoft, ver. 2203) and Prism9 (Graphpad, ver. 9.3.1). The *p*-values < 0.05 were considered to be significant.

## Results

**Fig 1:**
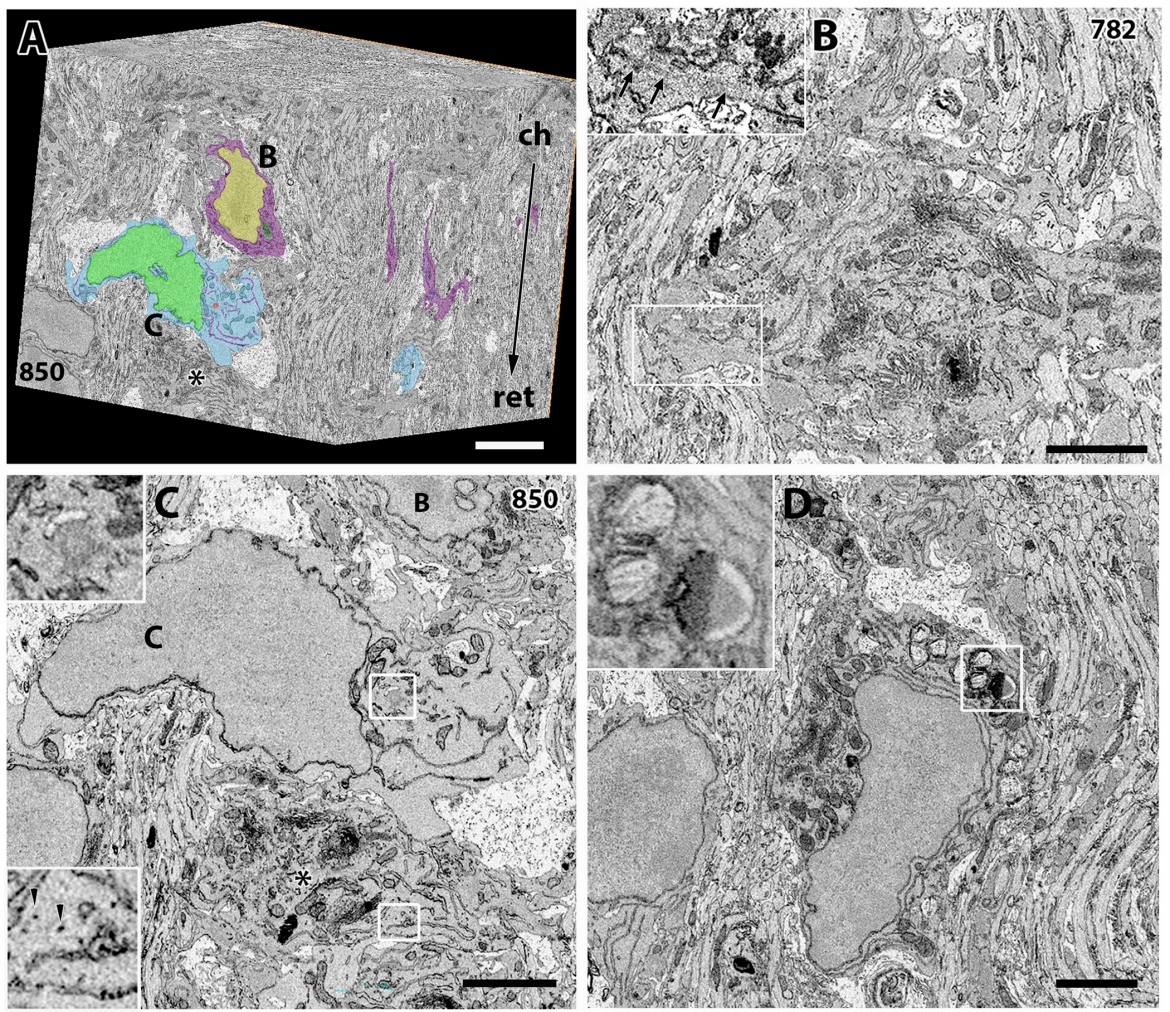
Glial cells imaged by SBF-SEM. (A) 3D reconstruction of a brain volume obtained from SBF-SEM images of the P4 optic nerve. An astrocyte with magenta color indicated by B is magnified in B, and a pOPC with light blue color indicated by C is magnified in C. An asterisk indicates another astrocyte magnified in C. Upper part of the cube block on the chiasmal side (ch) and the lower part on the retinal side (ret). The section number within the serial images was 850. (B) Higher magnification image of the astrocyte labeled as B in A, but 68 sections apart from A. The boxed area is magnified in the inset (upper left) and shows a bundle of intermediate filaments (arrows in the inset). Note the richness of cytoplasmic organelles in this astrocyte. (C) Higher magnification image of the pOPC labeled as C in A. Notably, the density of the cytoplasmic organelles in pOPC is much sparser compared with astrocytes (B) or microglia (D). A centriole within the boxed area of cell C is magnified in the upper inset. Another boxed area in the cell indicated by an asterisk contains glycogen granules and is magnified in the lower inset. (D) Higher magnification image of microglia. Note that dense bodies of lysosomes are contained in this cell and are magnified in the inset. Bars = 2 μm. B and C are magnified in S1 and S2 Figs, respectively.

Four image windows from two optic nerves were analyzed. More than 1000 serial images were collected from each image window, and therefore, nearly 5000 image frames were examined in total. From SBF-SEM, a complete series of multiple images can be analyzed (Fig 1A), and the presence of small structures, such as a cilium and filamentous structures, can be revealed, even if the structure is localized apart from the cell body (Fig 1B).

We identified astrocytes and microglia based on their ultrastructural characteristics in the serial images (Fig 1B and 1D). Astrocytes characteristically contained glycogen granules (Fig 1A, lower inset in Fig 1C, S1 Fig), and bundles of intermediate filaments were observed in the serial images of the same cell (Figs 1B and S2) [29]. Furthermore, glycogens were mainly localized in GLAST+ astrocytes and their progenitor cells (S3A Fig), while they were much less localized in the PDGFRα+ OPCs or excluded from Iba1+ microglia (S3B–S3D Fig). Microglia somas were identified using established criteria, including lysosomes, lipofuscin, and a long ER (Fig 1D) [19,30,31]. Fig 1 also shows a unique cell that contains relatively sparse cytoplasmic organelles and few or no glycogen granules, but no intermediate filaments (cell C in Fig 1, S1 and S2 Figs). Table 1 shows the number of cells that were examined. We observed 522 cells in four image windows. Among them, 404 cells showed astrocyte characteristics, and 14 cells exhibited microglia characteristics. Thirty-four cells could not be identified because most of their profiles extended beyond the SEM image border. We excluded these 452 cells from the list of possible OPCs. The remaining 70 cells, such as the cell C in Fig 1, were unique and morphologically distinct from astrocytes and microglia, as mentioned above (Figs 1, S1 and S2), and therefore, we regarded this type of cell as a putative OPC (pOPC). Although OPCs could not be strictly defined in this study, these identified cells represent the maximal potential distribution of OPCs in the optic nerve. Therefore, these 70 cells were analyzed as pOPCs. The fine structures of typical astrocytes and pOPCs are also shown in S4 Fig.

**Table 1:** The cell types and the number of cells observed.

Cell type	Cell number	%
OPC	70	13.4
Microglia	14	2.68
Astrocyte	404	77.1
Unidentified*	34	6.51
Total	522	

*These cells could not be identified because most of their profiles extended beyond the SEM image border.

### pOPCs

**Fig 2:**
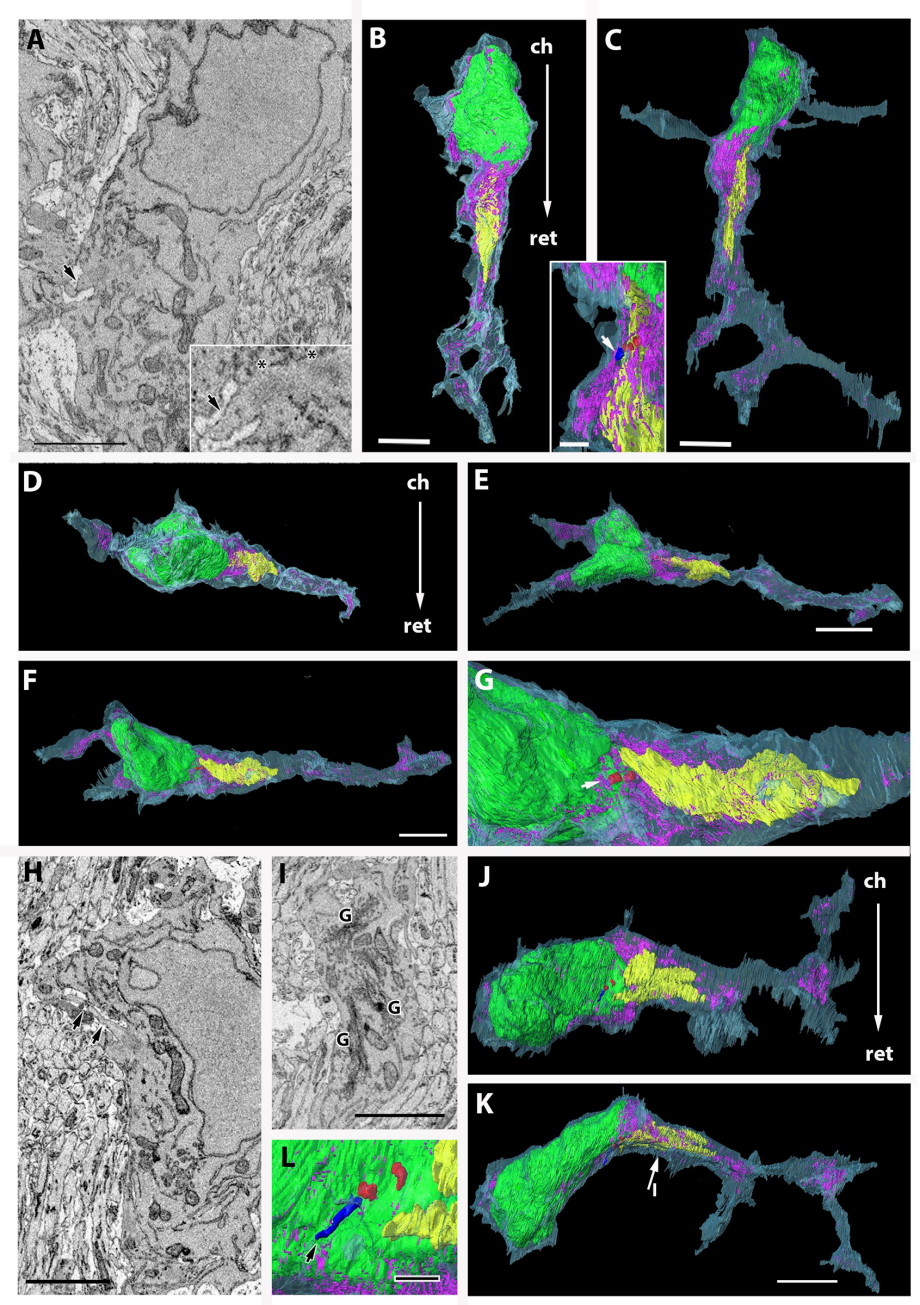
Fine structure and 3D reconstruction of pOPCs. Light blue, cytoplasm. Green, nucleus. Yellow, Golgi apparatus. Magenta, ER. Red, centrioles. Blue, cilium. (A–C) pOPC oriented along the optic nerve axons. (A) A pOPC in the SBF-SEM image. An arrow indicates a short cilium, which is magnified in the inset. A pair of centrioles (basal body) is indicated by asterisks in the inset. (B, C) 3D reconstruction of the pOPC, indicating that this cell is unipolar in shape, extends five processes, and contains a short cilium. Localization of the Golgi apparatus and ER is shown, most of which are close to the nucleus while a few ER are also observed in the distal part of the process. A 3D image of the short cilium is shown in the inset (arrow). This cell is magnified in S5 Fig. (D–G) 3D reconstructions of the pOPC in Fig 1C. (D–F) This cell is unipolar in shape with a single process and is arranged perpendicular to the array of optic nerve axons. (G) Perinuclear region of the cell is magnified. Golgi apparatus is composed of a single cisterna. Endoplasmic reticula are localized close to the nucleus and Golgi apparatus. A pair of centrioles are localized in the superficial part of the cell (arrow in G). (H–K) Another pOPC oriented perpendicular to the axon arrays. (H) This cell extends a relatively long cilium (arrows). Golgi apparatus can be observed in the proximal portion of the process (I). The 3D reconstruction reveals that this cell is unipolar in shape and extends two short processes. An arrow and I in K indicate an approximate region of cut plain in I. (L) 3D image of a cilium and basal body. Bars = 5 μm in B, C, F, and K; 2 μm in A, G, H, and I; 1 μm in L and the inset in C.

Inside each pOPC, the nucleus was surrounded by a thin cytoplasmic rim and a few organelles (Figs 1C and 2A, S1, S2 and S4).

ER, mitochondria, and Golgi apparatus were sparsely distributed in the cytoplasm (Figs 1C and S1), and the ER varied in length. All pOPCs contained a pair of centrioles, and sometimes, two pairs. Most centrioles were present close to the cell surface (Fig 2A, 2G, 2H and 2L). The SBF-SEM observations were consistent with light microscopy, where the γ-tubulin+ spots frequently overlapped with PDGFRα+ cell membranes in OPCs (S1F Fig), and the localization was distinct from that in astrocytes. The cilium, however, was observed in less than half of the pOPCs (45%, Table 2), and most of them were much shorter than those observed in astrocytes (Figs 2A and S5). Some centrioles without cilia were accompanied by flattened vesicles (upper inset in Fig 1C), which may indicate vesicular transport of centrioles toward the cell surface and early ciliogenesis.

**Table 2:** Types of centrioles and cilium.

Cell types	OPC	Astrocyte	Microglia
Types			
Ciliary pocket:			
Extending	0	291	0
Non-extending	0	23	0
Cilium from the cell surface	29	0	0
No cilium:			
Centrioles only	23	0	11
Ciliary vesicles	12	0	0
No cilium or centrioles	0	1	0

We reconstructed 3D images of 11 pOPCs, which revealed the localization of cytoplasmic organelles and cell contours (Fig 2, S1 Movie). ER and Golgi apparatus in these pOPCs tended to be localized close to the nucleus and the proximal part of the process (Fig 2B–2G and 2I–2K). The Golgi apparatus in most pOPCs was composed of a simple mass of cisterna (Fig 2D, 2F and 2G) with surrounding vesicles. In several pOPCs, the ER were localized in the distal part of the processes (Fig 2B, 2F and 2K), as well as in the perinuclear region. The lengths of cilia in the pOPCs were relatively short compared with those in astrocytes. The pOPCs in the P4 optic nerve were mostly unipolar in shape and did not extend more than six processes (Fig 1C). Their contours were relatively smooth, with a few short spines from the cell surface (Fig 2). Despite the unipolar shape of pOPCs, the directions of their processes were not uniform, with some extending toward the retinal direction (Fig 2B) and others extending perpendicularly toward the axon bundles (Fig 2D and 2J).

### Astrocytes

**Fig 3:**
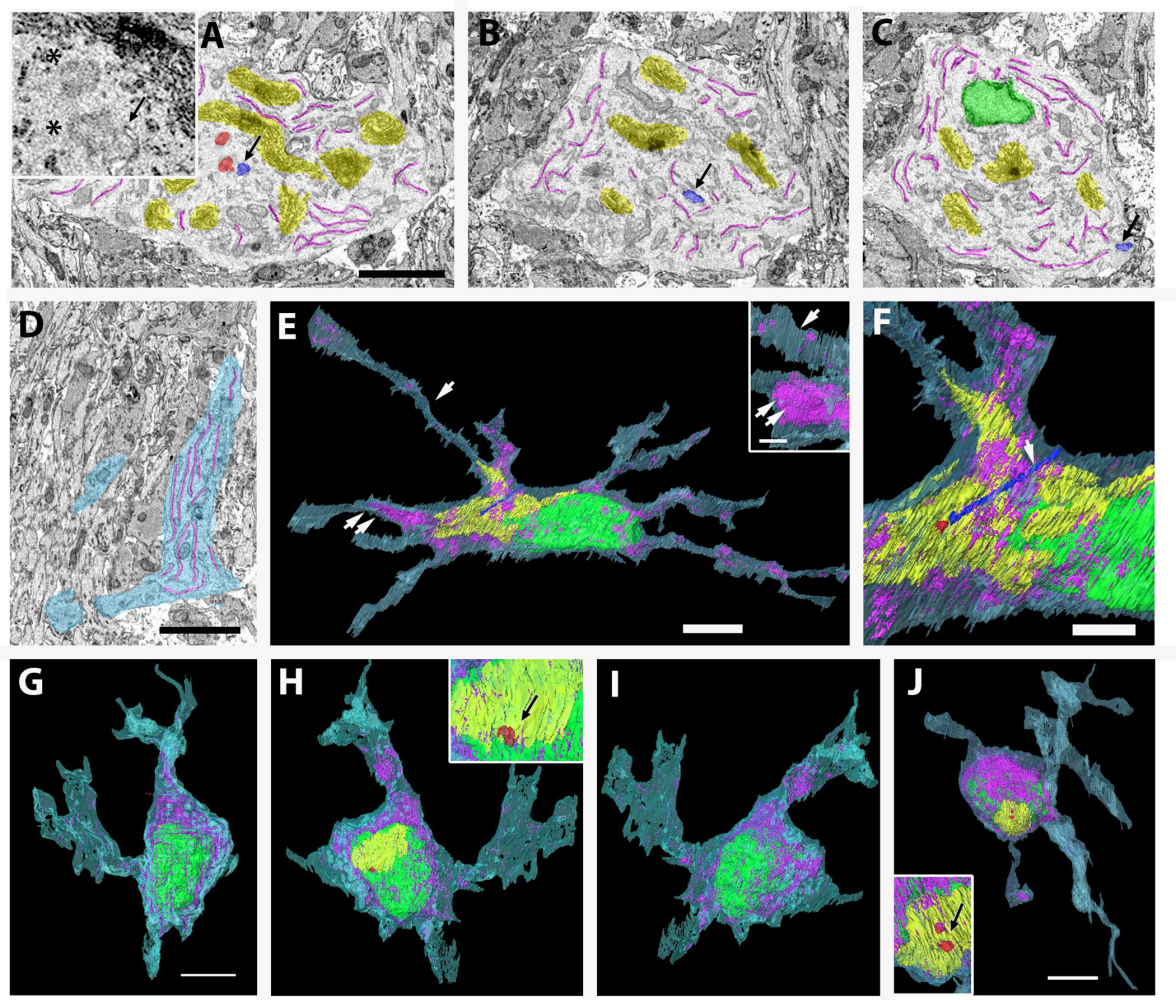
Fine structure and 3D reconstruction of astrocytes and microglia. (A–F) Astrocyte shown in Fig 1B. (A–C) To show the cilium clearly, the cytoplasm is colored in white, centrioles (basal bodies) in red, and the cilium in blue (arrows). Centrioles (asterisks) and the base of cilia are magnified in inset A. Note that the cilium shows a ciliary pocket. (D) ER in the proximal process. In this image, cytoplasm is colored in light blue. (E, F) A 3D reconstruction demonstrates that this cell extends 9–10 processes. Processes indicated by arrows are magnified in the inset at a different angle. Note that most processes are a flattened shape rather than cylindrical (compare E with the inset in E). Double arrows indicate an approximate region of cut plane in D. (F) Higher magnification image of the 3D reconstruction of the cilium (arrow). (G–J) 3D reconstructions of two microglia. (G–I) The microglia shown in Fig 1D contain lamellipodia at the process tips. The nucleus shows a disk shape, and ER are localized on one side of the cytoplasm. The inset in H shows a pair of centrioles closely associated with the Golgi apparatus. (J) The other microglia extend several processes without lamellipodia. The inset shows a higher magnification image of a pair of centrioles (red) closely positioned to the Golgi apparatus. Bars = 2 μm in A, D, F, and the inset in E, and 5 μm in E, G, and J.

Fig 3A–3F shows 2D and 3D images of a typical astrocyte.

Inside astrocytes, which contained intermediate filament bundles and glycogen granules, the ER were well developed and localized not only in the perinuclear region close to the Golgi apparatus (Fig 3A–3C) but also in the distal portion of the processes (Fig 3D and 3E). The Golgi apparatus was localized not only in the perinuclear region but also in the processes (Fig 3D and 3E, S1 and S2 Movies) and branched or lobulated into several small cisternae (Fig 3F). All astrocytes, except one, had pairs of centrioles that were accompanied by long cilia (Fig 3F). In contrast to pOPCs, the bases of cilia were invaginated deeply in the cytoplasm, forming a ciliary pocket (Fig 3A–3C, S3 Movie), and no cilium was extended from the cell surface in astrocytes. Rather, most of the cilia were extended from the deep part of the cytoplasm toward the outside of the cell, with the tips of short apical cilia exposed to the extracellular space (extending type). In addition, a small number of cilia in astrocytes were confined to the cytoplasm without extending to the outer space (non-extending intra-cytoplasmic type) (Table 2). Consistent with this, a pair of centrioles (basal bodies) were positioned in the deep part of the cytoplasm, and the basal bodies were closely associated with Golgi apparatus (Fig 3A). This observation was also confirmed by light microscopy, where immunoreactivity of γ-tubulin was observed in OPCs and astrocytes, but γ-butulin+ spots were localized close to the nuclei of GLAST+ astrocytes (S1E Fig), contrary to the PDGFRα+ cells described above. The profile of astrocytes was revealed by the 3D reconstruction of serial 2D images. Astrocytes in the newborn optic nerve extended more processes radially from the perikaryon than pOPCs (Fig 3E), and most processes had a flattened, not cylindrical, form. Fine spikes extended from the surface of cell bodies and processes (Fig 3E and 3F), and therefore, were more irregular and complicated.

### Microglia

**Fig 4:**
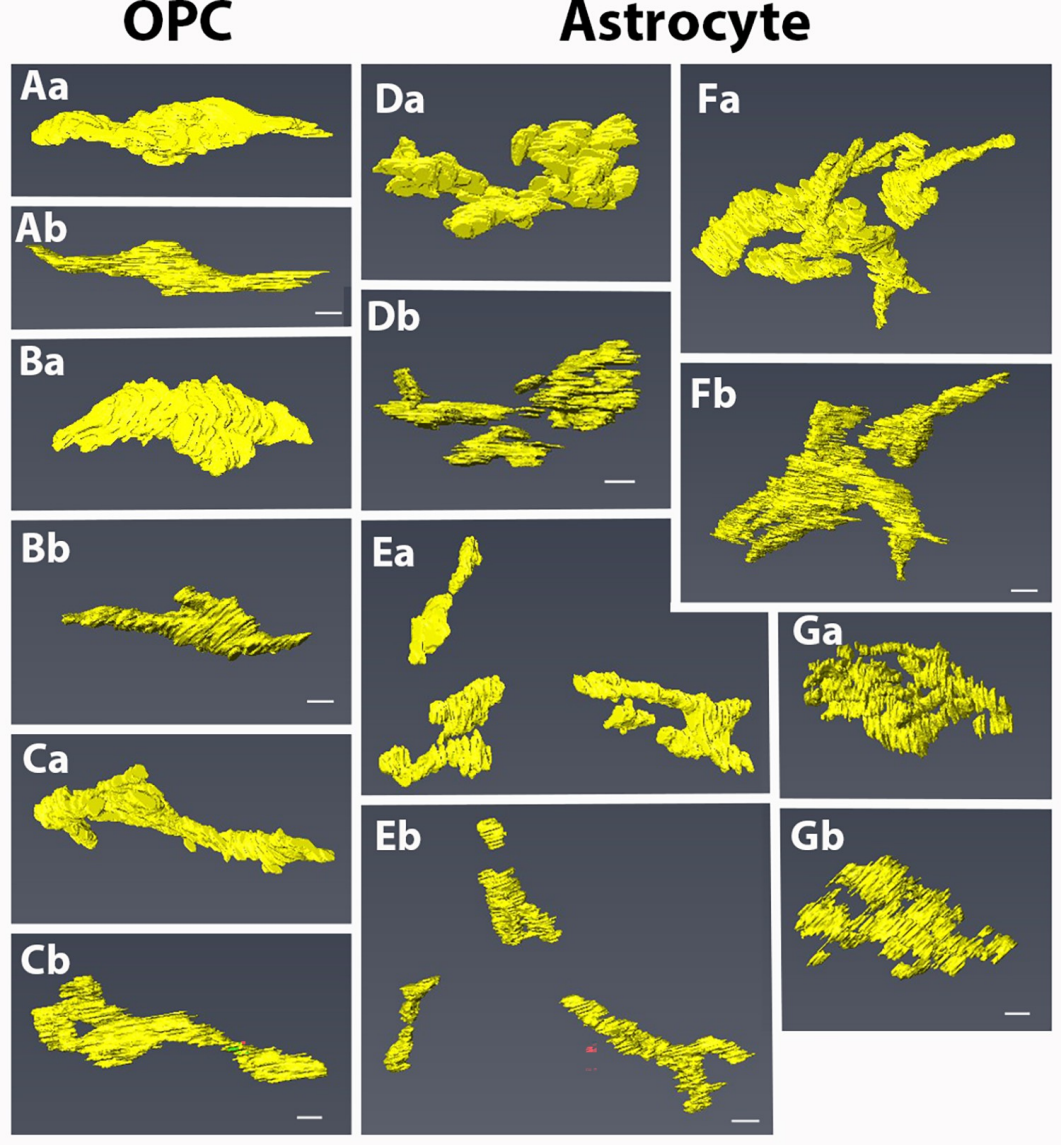
3D reconstruction of Golgi apparatus in OPCs (A–C) and astrocytes (D–G). a and b are the same Golgi apparatus viewed from different directions. Golgi apparatus in OPCs are composed of one or two cisternae while those in astrocytes are composed of several cisternae, sometimes separately distributed in the cell (E). Bars = 1 μm.

In this study, we identified a small number of typical microglia. The 3D reconstructions of two microglia are shown in Fig 3G–3J. The localization of ER on one side of the cytoplasm and the association of the ER with Golgi apparatus were observed. The microglia also contained a stack of large ER that were mostly localized on one side of the nucleus (Figs 1D and 3G–3J). In addition, Golgi apparatus were observed on the opposite side of ER (Fig 4H and 4J). All the observed microglia contained a pair of centrioles (Table 2) that were closely associated with Golgi apparatus (insets in Fig 3H and 3J), while none of the microglia had extended cilia (inset in Fig 4H and 4J).

### Comparison of ultrastructural profiles between pOPCs and astrocytes

We then compared cell profiles and ultrastructure of the OPCs with those of astrocytes, the most abundant cell type in the newborn optic nerve. We focused on the Golgi apparatus, cell contour, and cilium. The Golgi apparatus were three-dimensionally reconstructed from 2D images, and several samples are shown in Fig 4 and S1–S3 Movies.

**Fig 5:**
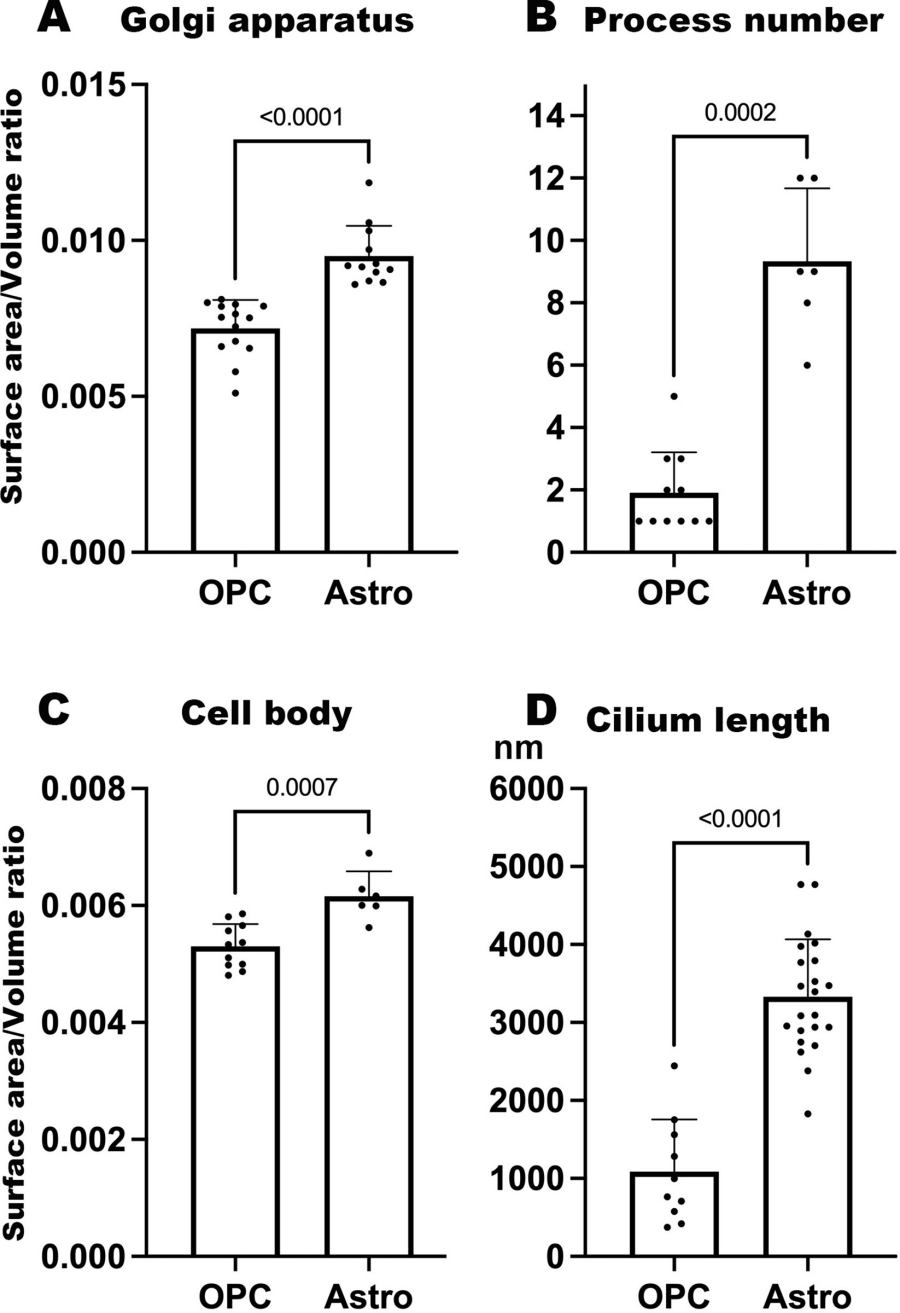
Quantitative analysis of differences between OPCs and astrocytes. (A) Ratio of the surface area and volume of Golgi apparatus. (B) Number of processes. (C) Ratio of the surface area to volume of a cell. (D) Length of the cilium in the pOPC and astrocyte. Values of A and C indicate unevenness in the surface of the structure. The *p*-values < 0.05 were considered to be significant.

The Golgi apparatus in pOPCs were composed of a single, or few, masses of cisterns positioned closely to the nucleus (Fig 4A–4C; S1 Movie). In contrast, the Golgi apparatus in astrocytes exhibited a branched shape and were composed of several masses of small cisterns (Fig 4D–4G). Additionally, the Golgi cisterns in astrocytes were distributed not only close to the nucleus but also in processes (Fig 4E; S2 and S3 Movies). The complexity of the Golgi apparatus shape was examined using a ratio of the surface area to volume (s/v ratio) because this ratio will be larger when the unevenness of the cell surface increases without an increase in volume. The s/v ratios of Golgi apparatus in astrocytes were significantly larger than those in pOPCs (Fig 5A).

Thus, the shape of Golgi apparatus in astrocytes was more complicated than that of Golgi apparatus in pOPCs.

Three-dimensionally reconstructed profiles of pOPCs and astrocytes revealed distinct numbers of processes. The pOPCs were mostly unipolar in shape while astrocytes extended several processes radially from the cell body. The average number of processes was greater in astrocytes than in pOPCs (Fig 5B). The complexity of cell contours was also compared between pOPCs and astrocytes using the s/v ratio, which was higher in astrocytes than in pOPCs (Fig 5C). Therefore, the 3D reconstruction profiles suggest that astrocytes have more uneven contours.

As mentioned above, all pOPCs and astrocytes contained a pair of centrioles. Cilium extension was observed in 45% of pOPCs, and the average length of the cilia, if any, was approximately 1 μm. In contrast, all astrocytes extended long cilia with average lengths of more than 3 μm (Fig 5D). Thus, the cilia were much longer in astrocytes than in pOPCs. In addition, the short cilia in the pOPCs were extended from the cell surface, while the long cilia in the astrocytes exhibited a ciliary pocket, and the centrioles were localized in close association with Golgi apparatus in the deep part of the cytoplasm. More than half (55%) of the pOPCs did not extend any cilia. Among them, 20% of the pOPCs contained small vesicles adjacent to the centrioles, as mentioned above (Table 2), which was not observed in astrocytes. The types of cilia and centrioles were summarized in Table 2 and schematically summarized in S6 Fig.

## Discussion

The present study revealed the ultrastructure of glial cells, especially OPCs, in the early postnatal optic nerve. We regarded cells that do not have specific characteristics of glial cells to be pOPCs [20]. Previous reports have also employed similar strategies to identify progenitor and precursor cells [32]. It is generally accepted that the lineage cells of astrocytes, including their progenitor cells, have glycogen granules [33,34]. A previous report demonstrated that proliferating astrocytes have intermediate filaments [35] and that astrocytes in the early postnatal optic nerve undergoing cell cycle progression express GFAP consistently [36]. Our immunohistochemical analysis showed that glycogens were abundant in GLAST+ astrocytes and their progenitor cells, and fewer glycogens were also localized in PDGFRα+ OPCs, but not in Iba1+ microglia (Fig 1). Glycogen is water soluble and flashed away from the tissue when washed in water for a long period [37,38]. In the present study, the cells regarded as pOPC contained few or no glycogen granules. The glycogen contents were probably decreased during washing with water. However, the astrocytes contained more glycogens than OPC (S3D Fig), and some glycogens remained in the astrocytes, even after prolonged washing with water (inset b in S4 Fig). In addition, the shapes of pOPCs characterized in the present study were similar to those in the early postnatal rat optic nerve [20,32]. Thus, although we did not use cell type-specific molecular markers, the pOPCs observed in this study are relevant to OPCs.

Notably, we identified unique ultrastructural characteristics of the pOPCs. The pOPCs in the P4 mice contained sparse cytoplasmic organelles, compared with astrocytes and microglia (Fig 1). Golgi apparatus and ER were mostly localized in the perinuclear regions (Fig 2), and the former were composed of a single mass of cisterna without extensive bifurcation or lobulation in the processes. All pOPCs contained a pair of centrioles in the submembranous cytoplasm. Cilium extension from the cell surface was observed in less than half of all pOPCs, while the remaining pOPCs did not show cilia (Table 2). Most pOPCs were unipolar in shape, and their outer cell surface was relatively smooth compared with that of astrocytes (Fig 5C). These cytological characteristics may reflect the cell function and activity of OPCs. The ER and Golgi apparatus are organelles responsible for the synthesis of membrane structures, including membrane proteins and lipids. pOPCs have a relatively simple profile, less uneven contours, and less process extension compared with astrocytes. Golgi apparatus in pOPCs have a simpler form with less lobulation and bifurcation (Figs 3 and 4) than those in astrocytes. The simple form of the Golgi apparatus may be indicative of less active production of membrane structure in pOPCs. In addition, the Golgi apparatus plays a role in the microtubule organizing center [39]. Astrocytes have more processes than pOPCs, which may require more cytoskeletal arrangement and result in a complicated form of the Golgi apparatus and their close association with centrioles.

The most prominent differences between pOPCs and astrocytes are the centrioles and cilium. Immunohistochemical analysis demonstrated the different localization of centrioles between astrocytes and pOPCs: γ-tubulin+ spots tended to overlap with PDGFRα+ cell membranes, while those in astrocytes tended to localize close to the nucleus (S3E and S3F Fig). SBF-SEM showed that all cilia in the astrocytes exhibit ciliary pockets, and thus, the basal body is localized in the deep part of the cytoplasm (Table 2, S6 Fig). In contrast, cilium extension was observed in 45% of the OPCs, and the average cilium length was significantly shorter than that in astrocytes (Fig 5B). All cilia in the pOPCs are extended from the cell surfaces (Table 2, S6 Fig), and therefore, the basal bodies of these cells were localized in the superficial parts. The remaining 55% of pOPCs did not extend a cilium. The primary cilium is reportedly the site of signal transduction from signals from outside the cell [40–42], and PDGFRα+ cells in the adult rat subventricular zone typically have a primary cilium [43]. Furthermore, nearly all pOPCs were found to contain centrioles in vitro, whereas there were far fewer that extended cilium (~16%), and some pOPCs undergo cilium retraction during the M phase of the cell cycle [44]. In the present study, centrioles accompanied by vesicles (i.e., ciliary vesicles) [24] were observed in pOPCs but not in astrocytes or microglia at this stage of the optic nerve. Ciliary vesicles may regulate the vesicular transport of the centrioles toward the superficial part of the cell [45]. Therefore, along with shorter cilium, these morphological characteristics suggest that the pOPCs observed in this study underwent ciliogenesis. In contrast, all astrocytes, except one, contained a long cilium with basal bodies (a pair of centrioles), which shows a ciliary pocket, whether the tip of the cilium was exposed to the outer space of the cell or not. This ciliary pocket has been observed not only in astrocytes (present study) but also in migrating neurons in the brainstem [46] and cerebellum [47]. In the present study, the state of maturation may be different among three glial types in the P4 optic nerve. Astrocytes and microglia may be more mature than pOPCs because they have more cytoplasmic organelles, which may be related to the ciliary formation and cilium types. Although ciliary vesicles are not a specific structure in pOPCs [46], they can be a selective morphological marker for OPCs in the early postnatal optic nerve. Moreover, the ciliary pocket and ciliary vesicles may be too small to conduct real-time imaging analysis, but future developments may uncover unique dynamics of cilia and their related structures.

Recently, subpopulations of OPCs have been reported with respect to sites of origins, stages of generation, and expressions of functional molecules [48–51]. OPCs arise from multiple restricted neuroepithelial layers during development. For example, in the spinal cord, the majority of OPCs and OLs are generated in the ventral ventricular zone in early development, and the dorsal neuroepithelial layer produces fewer OPCs in later stages [6]. Thus, the OLs in the spinal cord have two subpopulations with respect to the sites of origin, and both subpopulations show similar electrophysiological properties. In the optic nerve, although OLs have an origin in the preoptic area in the fetal stages [2], it is still unclear whether the preoptic area is the only origin for OLs in the optic nerve. Our results concerning the fine structures of pOPCs mostly agree with the previous reports. Moreover, pOPCs can be subdivided into two groups. One group of pOPCs extends a short cilium from the cell surface, while the other does not show one. This may be indicative of ciliogenesis because of the short length of the cilia and the presence of vesicles. However, at the ultrastructural level, we could not recognize a subpopulation of pOPCs.

As previously discussed [20], “small and large progenitor” cells and “neuroglial precursors” [52,53] show similarities with glial progenitor cells in the early postnatal optic nerve. In the present study, we additionally described the internal ultrastructure of those precursor cells, including the distribution of organelles, cilium, and centrioles, as well as whole cell contours. Fewer cytoplasmic organelles and a simpler form (unipolar in shape) were common features of cells in the immature or developing stages [54]. We observed only a single stage (P4) of the optic nerve. However, OPCs gradually change their morphology, such as the direction and number of process extensions [55–57], and the fine structures of OPCs may change during their development. Further SBF-SEM studies of glial cells in various developmental stages will elucidate the detailed developmental processes of cellular morphogenesis in OPCs.

## Conclusion

In the present study, we examined the ultrastructure of OPCs in the early postnatal mouse optic nerve by SBF-SEM. The 2D pictures and 3D reconstructed profiles revealed the shapes and distributions of glial cells and their cytoplasmic organelles, which were compared between pOPCs and astrocytes, the most abundant cell type in the optic nerve. pOPCs contained relatively sparse organelles such as ER and Golgi apparatus. The Golgi apparatus in pOPCs was relatively simple and composed of a single mass of cisterna, whereas that in astrocytes exhibited a bifurcated form. Although the pOPCs, astrocytes, and microglia all contained centrioles, cilium formation was observed in less than half of pOPCs, whereas nearly all astrocytes extended a cilium with a ciliary pocket. Fewer processes were extended from pOPCs, and their cell contours were relatively simple. Overall, the ultrastructural characteristics of pOPCs were unique compared with other glial cells such as astrocytes and microglia.

## Supporting information

S1 Fig Higher magnification image of Fig 1A. Fig 1A is magnified to show details of typical astrocytes (B, *) and a pOPC (C). The most striking difference between astrocytes and pOPCs in this picture is the richness of cytoplasmic organelles. A boxed area in cell * is magnified in the inset. Arrow heads indicate possible glycogen granules. Bar = 5 μm.
(TIF)

S2 Fig Higher magnification image of Fig 1B. Fig 1B is magnified to show details of astrocytes. Cells B and C in this picture correspond to those in Fig 1. A boxed area is magnified in the inset. Arrow heads indicate bundles of filamentous structures. Bar = 5 μm.
(TIF)

S3 Fig Localization of glycogen granules and γ-tubulin in glial cells of the P4 optic nerve. (A–C) Epifluorescent photomicrographs of glycogen localization in the newborn mouse optic nerve. Double immunofluorescence with anti-Glycogen antibody, together with anti-GLAST (A, astrocyte), with anti-PDGFERa (B, OPC), and with anti-Iba1 (C, microglia) antibodies. Note that most, if not all, glycogens are localized on GLAST+ astrocytes. (D) Glycogen immunoreactive dots number on each glial type. The counted cell numbers: GL, GLAST+ cell, 67 cells; Ra, PDGFRα+ cell, 63 cells; Iba, Iba1+ cell, 20 cells. ****, *p* < 0.0001. E and F, confocal laser scanning photomicrographs of γ-tubulin localization in GLAST+ astrocytes (E), and in PDGFRα+ OPC (F). Note that γ-tubulin+ spots in GLAST+ astrocytes are localized close to the nucleus while those in PDGFRα+ OPCs are frequently overlapped with the cell membrane. Bars = 20 μm.
(TIF)

S4 Fig 2D image of typical astrocytes and a pOPC. The cell of A is a typical pOPC. Thin cytoplasmic rim, surrounding nucleus, contains sparse cytoplasm. Insets A1–A3 demonstrate a pair of centrioles (arrow heads) in the leading process of cell A, localized 6 μm apart from this image. Cells B and C are typical astrocytes, containing intermediate bundles (asterisk in inset B) and glycogen granules (arrow heads in inset B). The boxed area in C demonstrates a ciliary pocket with a basal body, whose serial images are magnified in insets C1–C4. Bar = 5 μm.
(TIF)

S5 Fig Higher magnification images of pOPC shown in Fig 2E. The right image shows the same cell in Fig 2A. Arrow indicates a short cilium that is magnified in the left column. (G) Golgi apparatus in the leading process. The left column shows the serial images of the short cilium (arrows) and its basal body (asterisks) at 72-nm intervals. Scale bar = 2 μm.
(TIF)

S6 Fig Schema of cilium/centriole types in the glial cells in the newborn optic nerve. Table 2 is summarized in the schematic illustrations. Number in the parentheses is the number of cells with that cilium type per number of glial subtypes.
(AI) Click here for additional data file.

S1 Movie This movie shows the pOPC, shown in Fig 2A–2D, and a Golgi apparatus in Fig 4B. Light blue, cytoplasm. Green, nucleus. Yellow, Golgi apparatus. Magenta, ER. Red, centrioles. Blue, cilium (not present in this cell).
(MP4)

S2 Movie This movie shows the astrocyte, from which the Golgi apparatus is shown in Fig 4E. This cell contains no cilia.
(MP4)

S3 Movie This movie shows the astrocyte shown in Fig 3A–3F and a Golgi apparatus in Fig 4F. Cytoplasm is indicated by white color.
(MP4)
