# Supplementary figures and images for: Ultrastructural characteristics of oligodendrocyte precursor cells in the early postnatal mouse optic nerve observed by serial block-face scanning electron microscopy
Source: PLoS One. 2022 Dec 1;17(12):e0278118. doi: 10.1371/journal.pone.0278118 (PMC9714907; doi:10.1371/journal.pone.0278118)

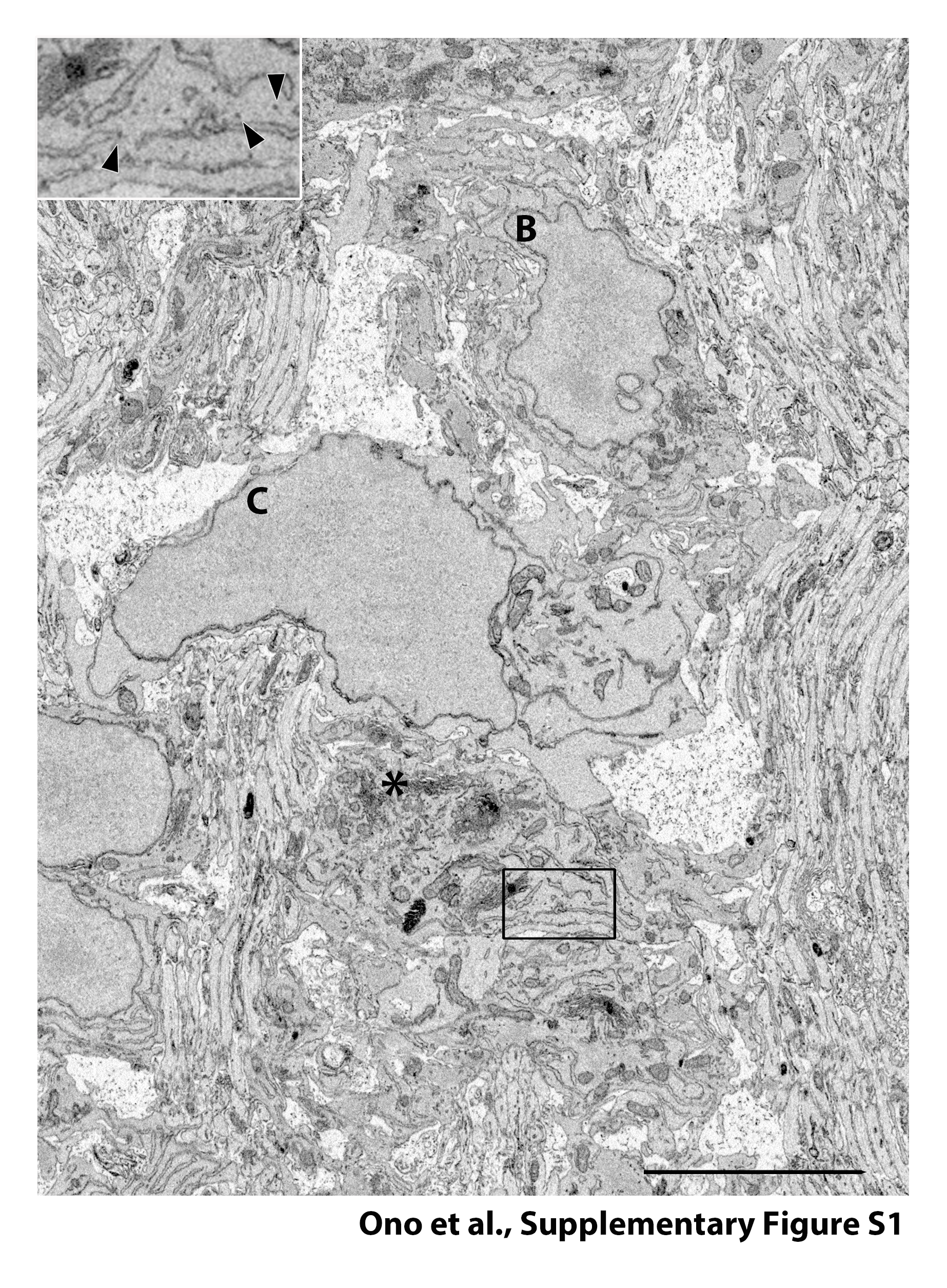

Supplement: S1 Fig — Fig 1A is magnified to show details of typical astrocytes (B, *) and a pOPC (C). The most striking difference between astrocytes and pOPCs in this picture is the richness of cytoplasmic organelles. A boxed area in cell * is magnified in the inset. Arrow heads indicate possible glycogen granules. Bar = 5 μm. (TIF) [file pone.0278118.s001.tif]

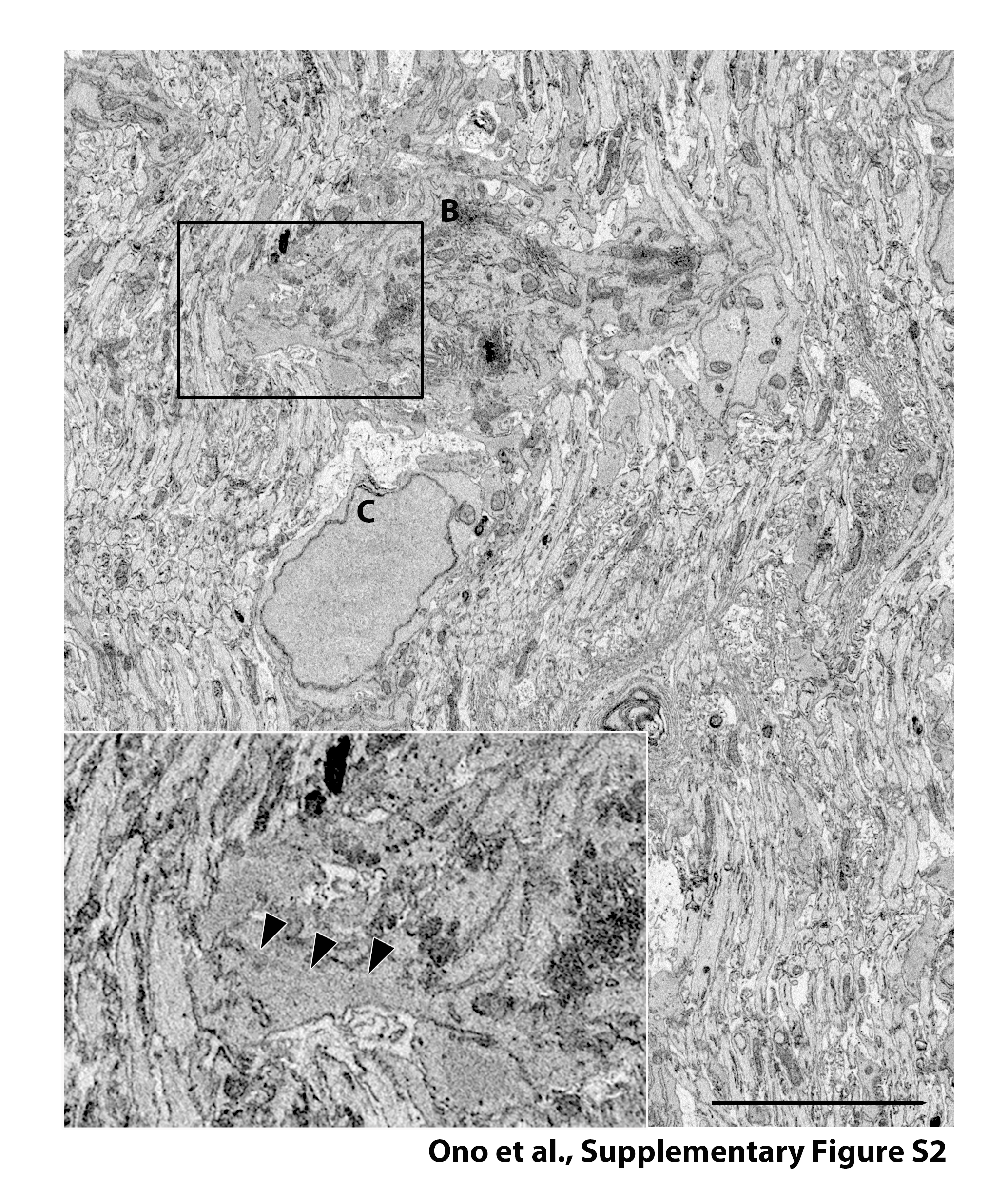

Supplement: S2 Fig — Fig 1B is magnified to show details of astrocytes. Cells B and C in this picture correspond to those in Fig 1. A boxed area is magnified in the inset. Arrow heads indicate bundles of filamentous structures. Bar = 5 μm. (TIF) [file pone.0278118.s002.tif]

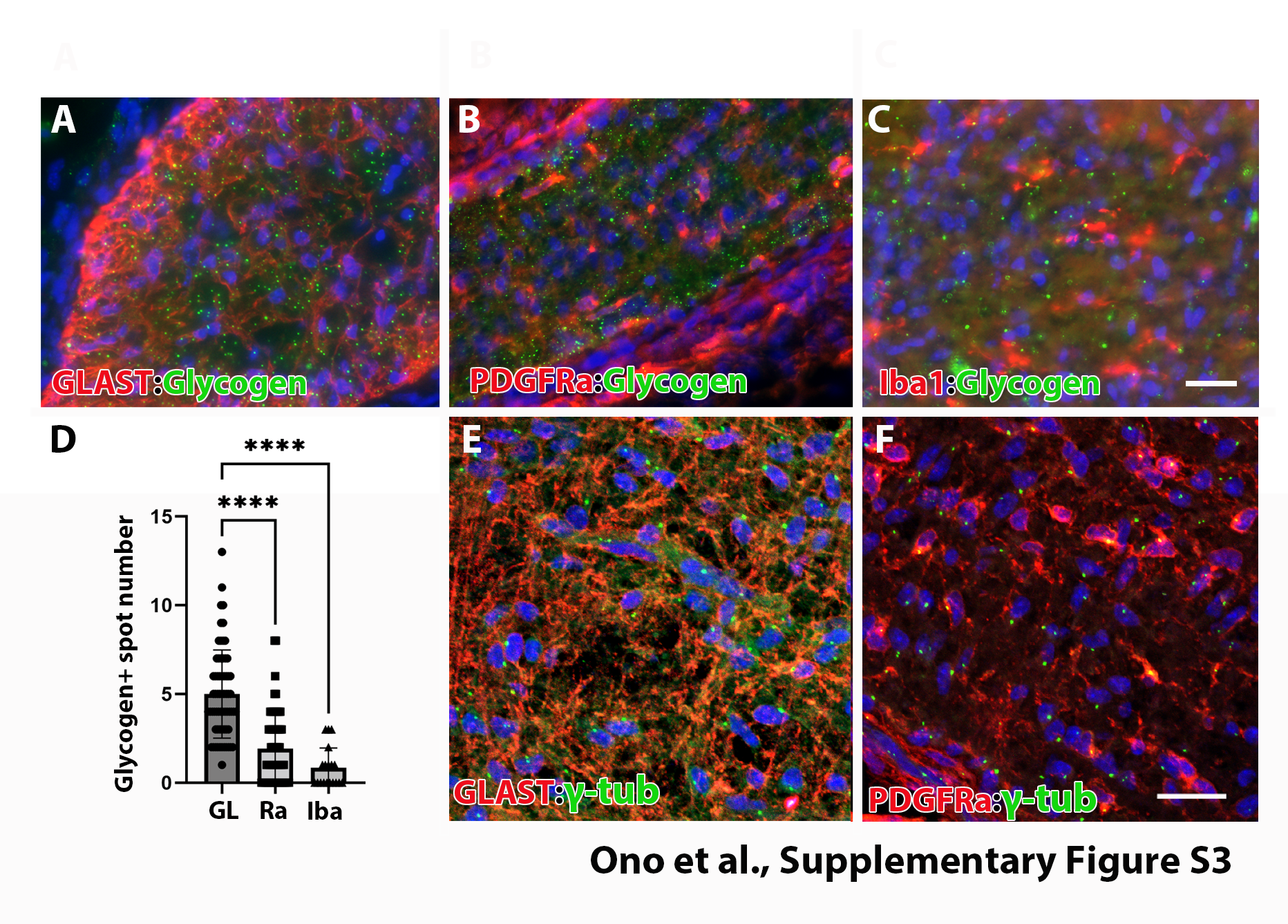

Supplement: S3 Fig — (A–C) Epifluorescent photomicrographs of glycogen localization in the newborn mouse optic nerve. Double immunofluorescence with anti-Glycogen antibody, together with anti-GLAST (A, astrocyte), with anti-PDGFERa (B, OPC), and with anti-Iba1 (C, microglia) antibodies. Note that most, if not all, glycogens are localized on GLAST+ astrocytes. (D) Glycogen immunoreactive dots number on each glial type. The counted cell numbers: GL, GLAST+ cell, 67 cells; Ra, PDGFRα+ cell, 63 cells; Iba, Iba1+ cell, 20 cells. ****, p < 0.0001. E and F, confocal laser scanning photomicrographs of γ-tubulin localization in GLAST+ astrocytes (E), and in PDGFRα+ OPC (F). Note that γ-tubulin+ spots in GLAST+ astrocytes are localized close to the nucleus while those in PDGFRα+ OPCs are frequently overlapped with the cell membrane. Bars = 20 μm. (TIF) [file pone.0278118.s003.tif]

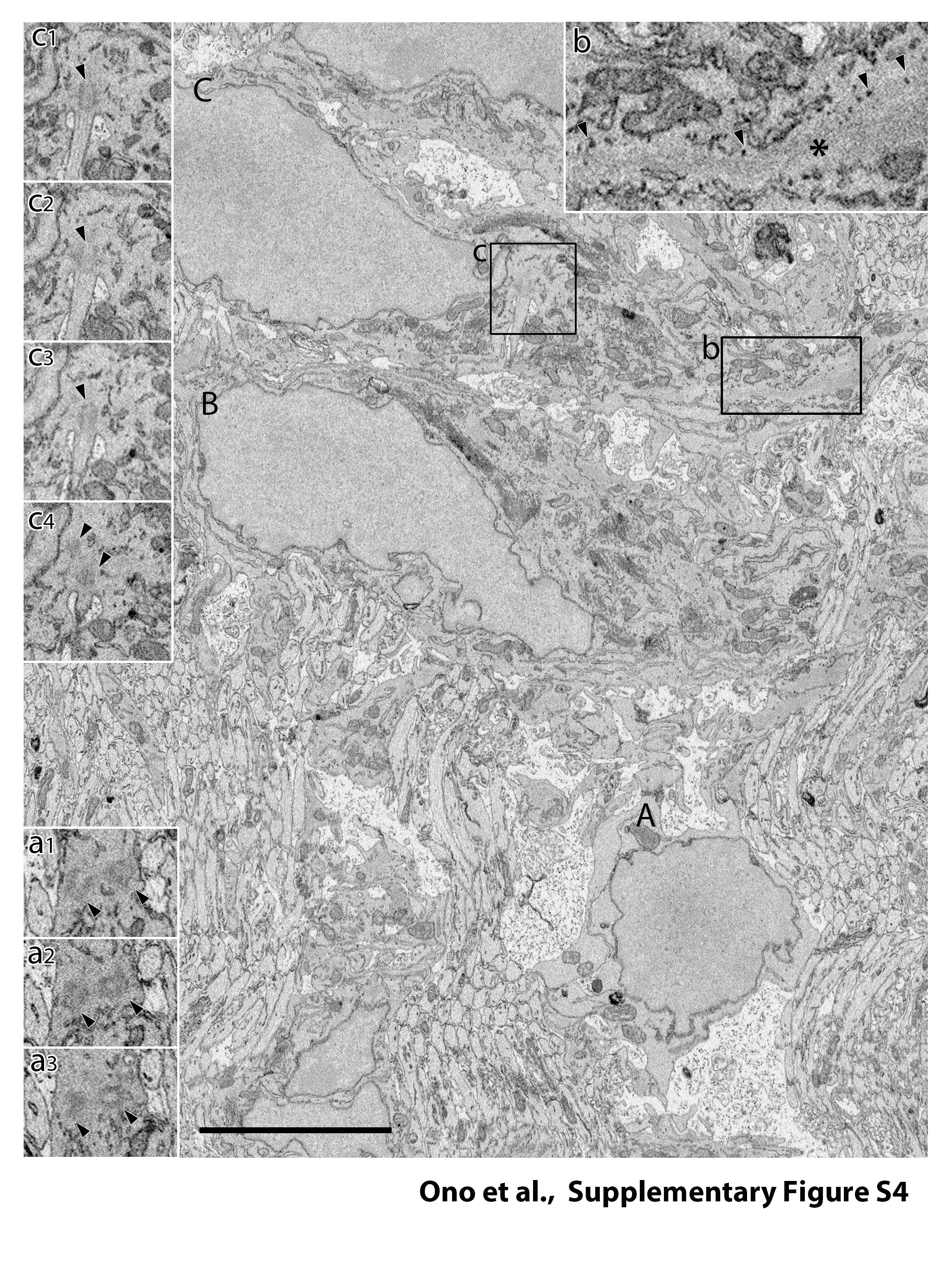

Supplement: S4 Fig — The cell of A is a typical pOPC. Thin cytoplasmic rim, surrounding nucleus, contains sparse cytoplasm. Insets A1–A3 demonstrate a pair of centrioles (arrow heads) in the leading process of cell A, localized 6 μm apart from this image. Cells B and C are typical astrocytes, containing intermediate bundles (asterisk in inset B) and glycogen granules (arrow heads in inset B). The boxed area in C demonstrates a ciliary pocket with a basal body, whose serial images are magnified in insets C1–C4. Bar = 5 μm. (TIF) [file pone.0278118.s004.tif]

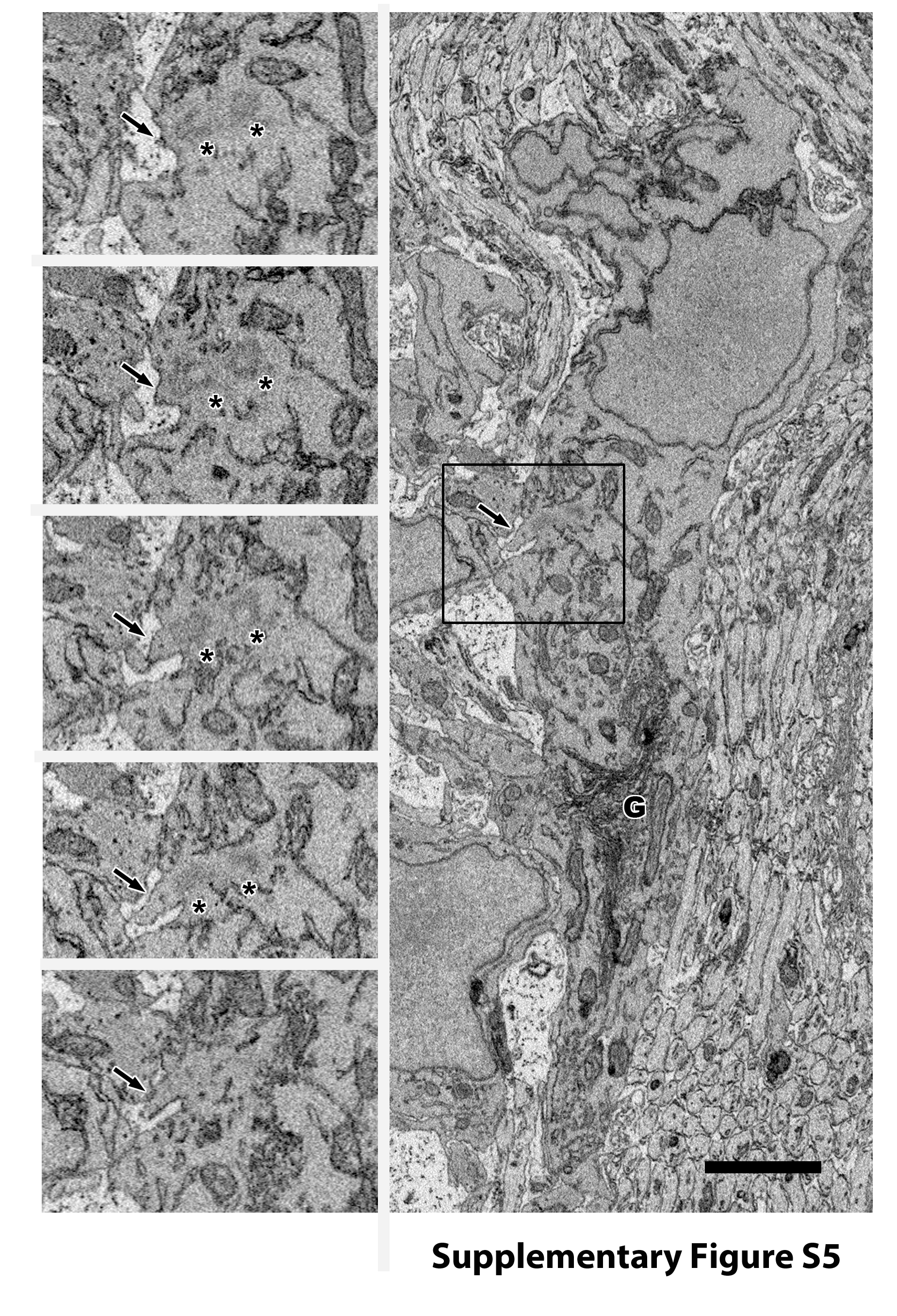

Supplement: S5 Fig — The right image shows the same cell in Fig 2A. Arrow indicates a short cilium that is magnified in the left column. (G) Golgi apparatus in the leading process. The left column shows the serial images of the short cilium (arrows) and its basal body (asterisks) at 72-nm intervals. Scale bar = 2 μm. (TIF) [file pone.0278118.s005.tif]
